# The *Arabidopsis thaliana* Brassinosteroid Receptor (AtBRI1) Contains a Domain that Functions as a Guanylyl Cyclase *In Vitro*

**DOI:** 10.1371/journal.pone.0000449

**Published:** 2007-05-23

**Authors:** Lusisizwe Kwezi, Stuart Meier, Lyndon Mungur, Oziniel Ruzvidzo, Helen Irving, Chris Gehring

**Affiliations:** 1 Department of Biotechnology, University of the Western Cape, Bellville, South Africa; 2 Department of Pharmaceutical Biology, Victorian College of Pharmacy, Monash University, Melbourne, Victoria, Australia; Purdue University, United States of America

## Abstract

**Background:**

Guanylyl cyclases (GCs) catalyze the formation of the second messenger guanosine 3′,5′-cyclic monophosphate (cGMP) from guanosine 5′-triphosphate (GTP). Cyclic GMP has been implicated in an increasing number of plant processes, including responses to abiotic stresses such as dehydration and salt, as well as hormones.

**Principle Findings:**

Here we used a rational search strategy based on conserved and functionally assigned residues in the catalytic centre of annotated GCs to identify candidate GCs in *Arabidopsis thaliana* and show that one of the candidates is the brassinosteroid receptor AtBR1, a leucine rich repeat receptor like kinase. We have cloned and expressed a 114 amino acid recombinant protein (AtBR1-GC) that harbours the putative catalytic domain, and demonstrate that this molecule can convert GTP to cGMP *in vitro*.

**Conclusions:**

Our results suggest that AtBR1 may belong to a novel class of GCs that contains both a cytosolic kinase and GC domain, and thus have a domain organisation that is not dissimilar to that of atrial natriuretic peptide receptors, NPR1 and NPR2. The findings also suggest that cGMP may have a role as a second messenger in brassinosteroid signalling. In addition, it is conceivable that other proteins containing the extended GC search motif may also have catalytic activity, thus implying that a significant number of GCs, both in plants and animals, remain to be discovered, and this is in keeping with the fact that the single cellular green alga *Chlamydomonas reinhardtii* contains over 90 annotated putative CGs.

## Introduction

Guanylyl cyclases (GCs) have been identified in many diverse prokaryotes and eukaryotes where they catalyse the formation of the second messenger guanosine 3′,5′-cyclic monophosphate (cGMP) from guanosine 5′-triphosphate (GTP) [1]. In higher plants cGMP has been shown to act as a second messenger in a large number of physiological responses [2], [3] that include cGMP-mediated changes of the transcriptome [4], NO-dependent signaling [5] as well as gravitropic responses [6] and plant hormone-dependent responses [7]–[9]. Furthermore, significant and transient increases in intracellular cGMP levels have also been reported in response to plant natriuretic peptides (PNPs) [8], [10] as well as NaCl and drought stress [11]. The first functional GC in higher plants was identified with a search motif based on several functionally assigned amino acids in the catalytic domain of known GCs from lower eukaryotes and animals [12].

Here we show that a rationally designed search motif of the catalytic domain identifies several members of the family of leucine rich repeat receptor-like kinases (LRR RLKs) including an *Arabidopsis thaliana* brassinosteroid receptor (AtBRI1). A recombinant domain protein was made which tested positive for GC activity *in vitro.* The implications of this finding for both the projected number of different classes of GCs and the role of cGMP in brassinosteroid signaling are discussed.

## Results

### Extending the search GC search motif and identification of AtBRI1

**Figure 1:**
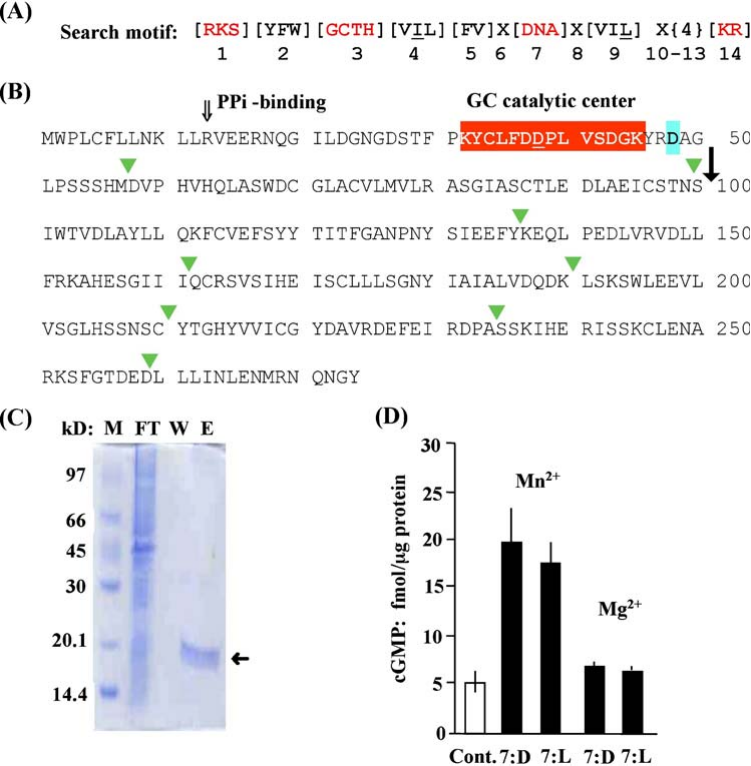
Site-directed mutagenesis and functional testing of AtGC1(1–100). (A) Original 14 amino acid search motif for GCs [12]; substitutions are in square brackets, X represents any amino acid and curly brackets define the number of amino acids. (B) AtGC1 (At5g05930): The position of the GC catalytic centre is marked in red; the underlined aspartic acid [D] is the amino acid that has been changed into a leucine [L] by site directed mutagenesis. The open arrow marks the conserved PPi-binding arginine [R] and the C-terminal putative metal binding site is highlighted in aquamarine. The green triangles point to exon borders and the solid arrow shows the border of the fragment that we have tested for GC activity. (C) SDS-PAGE of the 3 purification steps of the recombinant protein AtGC1(1–100); “M” is the molecular weight marker, “FT” is the protein in the flow-through, “W” is the wash and “E” is the eluted recombinant protein. (D) *In vitro* GC activity assay. The control (cont.; empty bar) was obtained by omitting protein in the reaction mix and the concentration of GTP was 1mM and that of Mn^2+^ or Mg^2+^ was 5 mM. The values for the wild-type protein (N-terminal fragment of 100 amino acids containing a [D] in position 7 of the catalytic centre) and the mutated protein (N-terminal fragment of 100 amino acids containing [L] in position 7 of the catalytic centre) are represented with solid bars. The bar values represent the mean cGMP (+/−SEM) generated in 15 minutes in three samples and the response pattern is representative of 3 independent experiments.

**Figure 2:**
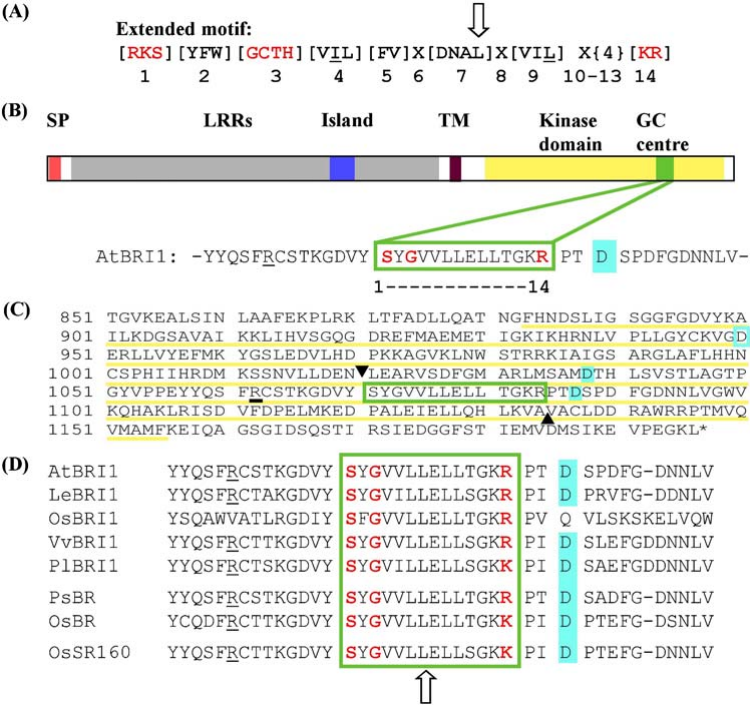
Structural features of the GC catalytic domain and the *Arabidopsis thaliana* brassinosteroid receptor AtBRI1. (A) The 14 amino acid long original search motif (modified after [12] with an inclusion of [L] in position 7). Red amino acids are functionally assigned residues of the catalytic centre. The residue in position 1 does the hydrogen bonding with the guanine, the amino acid in position 3 confers substrate specificity and the residues in positions 10 and 14 stabilise the transition (GTP/cGMP). (B) Representation of the domain organisation of AtBRI1 containing a signal peptide (SP), leucine rich repeats (LLRs) including an island, a transmembrane domain (TM), a GC centre embedded in the kinase domain. The position (16 or 17) outside the catalytic centre is implicated in Mg^2+^/Mn^2+^-binding (aquamarine). (C) Amino acid sequence of the intracellular C-terminal region of AtBRI1. The kinase domain is underlined (yellow), the GC domain is boxed in green, putative Mg^2+^/Mn^2+^-binding sites are highlighted (aquamarine), the proposed PPi binding is underlined in black, and the recombinant protein (AtBRI1-GC) tested for GC activity *in vitro* is delineated by solid triangles (σ). (D) Alignment of AtBRI1-like sequences. AtBRI1 (At4g39400), LeBRI1 (tomato|TC185049, Q9LJF3), OsBRI1 (Os06g0691800), VvBRI1 (grape |TC70352, Q9ZWC8), PlBRI1 (poplar|TC57820, Q9ZWC8), PsBR (BAC99050), OsBR (Os08g25380), OsSR160 (BAD34326.1, AP006156.2).

The original GC search motif [RKS][YFW][GCTH][VIL][FV]X[DNA]X[VIL]X{4}[KR] [12] (Figure 1A) yielded seven Arabidopsis candidate proteins including AtGC1 that has been demonstrated to have GC activity *in vitro* [12]. Two of seven proteins are annotated kinases and one of the two (At1g79680) belongs to the group of wall associated kinase-like proteins (WAKLs). In a quest to identify further candidate GCs in plants we mutated the position 7 in the original search motif. This position is assigned as having a role in dimerisation that may not be a critical requirement for GC functionality. When [D] in position 7 is substituted by [L] in a 100 amino acid recombinant AtGC1(1–100), a domain that is encoded by the first exon of AtGC1 (Figure 1B), no significant loss in catalytic GC activity occurs (Fig. 1D). This implies firstly that a 100 amino acid domain is sufficient for activity and secondly, that [D] in position 7 is not essential for AtGC1 GC activity. These observation may hold for a number of GCs. Consequently we added [L] to make position 7 [DNAL] (Fig. 2A). This extended motif retrieves 123 *Arabidopsis thaliana* proteins including the brassinosteroid receptor AtBRI1 (*Arabidopsis thaliana* brassinosteroid insensitive 1 (NP_195650.1, At4g39400.1). Furthermore, since the catalytic asparagine [N] in position 10, while quite conserved in some classes of nucleotide cyclases is substituted by [AILSTYHDE] in many annotated GCs [13], we further modified the motif to [RKS][YFW][GCTH][VIL][FV]X{3}[VIL]X{4}[KR]. All three motifs are specific for GC rather than adenylyl cyclases (ACs) since they contain the residues [GCTH] in position 3 facing the purine and determining substrate specificity for GTP rather than ATP [13]–[15] and GenBank contains no annotated ACs that conform to either of the motifs.

The third and most relaxed motif appears in 171 Arabidopsis proteins of which 88 contain [EQCTDRVHY] at position 16 or 17 which is presumed responsible for metal ion binding [13]. Metal binding is normally associated with aspartic or glutamic acid ([DE]). Within the group of 88 proteins; 27 contain a PPi-binding [R] between 5 and 20 amino acids N-terminal of the core motif.

These 27 Arabidopsis proteins (Table 1) represent a highly significant (p<1e^−5^) enrichment for the gene ontology GO categories of phosphorus metabolic processes, protein metabolic process, cellular macromolecular metabolic process and biopolymer metabolic process. It is noteworthy that several are annotated as leucine rich repeat receptor like kinases (LRR RLKs) including AtBRI1 (Fig. 2). In AtBRI1, the putative catalytic core was identified within the cytosolic kinase domain.

**Table 1:** Unique Arabidopsis proteins retrieved with the search pattern: [R]X{5,20}[RKS][YFW][GCTH][VIL][FV]X{3}[VIL]X{4}[KR]X{1,2}[D]

I.D.	Annotation
At1g09050	Function unknown
At1g14370	Protein kinase APK2a
At1g17750	Leucine-rich repeat transmembrane protein kinase
At1g28440	Leucine-rich repeat transmembrane protein kinase
At1g48220	Serine/threonine protein kinase, similar to Pto kinase interactor
At1g69270	Leucine-rich repeat family protein, protein kinase family
At1g69910	Protein kinase family protein
At1g73080	Nucleotide binding leucine-rich repeat RK, immune response
At1g76370	Protein kinase
At2g01860	Pentatricopeptide (PPR) repeat-containing protein
At2g02800	Protein kinase APK2b
At2g26330	Leucine-rich repeat protein kinase, putative ERECTA
At2g32800	Kinase family protein with dual protein kinase domains
At3g02130	Leucine-rich repeat transmembrane protein kinase
At3g02810	Protein kinase family protein
At3g07070	Protein kinase family protein
At3g24790	Protein kinase family protein
At3g46340	Leucine-rich repeat protein kinase, similar to light repressible receptor PK
At3g46350	Leucine-rich repeat protein kinase
At3g46400	Leucine-rich repeat protein kinase, similar to light repressible receptor PK
At4g20270	Leucine-rich repeat transmembrane PK, CLAVATA1 receptor kinase
At4g39400	BRI1 (ATBRI1-GC)
At5g05930	AtGC1
At5g07180	Leucine-rich repeat protein kinase, putative ERECTA
At5g10530	Lectin protein kinase, similar to receptor lectin kinase 3
At5g16500	Protein kinase family protein
At5g62230	Leucine-rich repeat protein kinase, putative ERECTA

PK, protein kinase; RK, receptor kinase. Note: The listed proteins constitute a significant overrepresentation (p<1e^−5^) in the Fatigo+ (level 4) categories of phosphorus metabolic processes, protein metabolic process, cellular macromolecular metabolic process and biopolymer metabolic process.

The choice of for further testing AtBRI1 was informed by several factors including the fact that brassinosteroids are physiologically well characterised growth regulators that await further elucidation of their signal transduction networks as well as the availability of a number of AtBRI1 mutants that can support these investigations. Brassinosteroid receptors have been identified in several other species and these also contain the conserved GC motif (Fig. 2D).

The recombinant protein that we decided to synthesise and test for *in vitro* activity contains the predicted GC catalytic centre of AtBRI1 (At4g39400) and 50 additional amino acids on both the N- and the C-terminus (Fig. 2C). This peptide (AtBRI1-GC) is part of the cytoplasmic domain containing the N-terminal part aspartic acid ([D] at −33 from the catalytic centre) implicated in metal binding [16] as well as a metal binding [D] in position 17 relative to the C-terminus of the motif.

### Testing a recombinant GC domain (AtBRI1-GC) for activity

**Figure 3:**
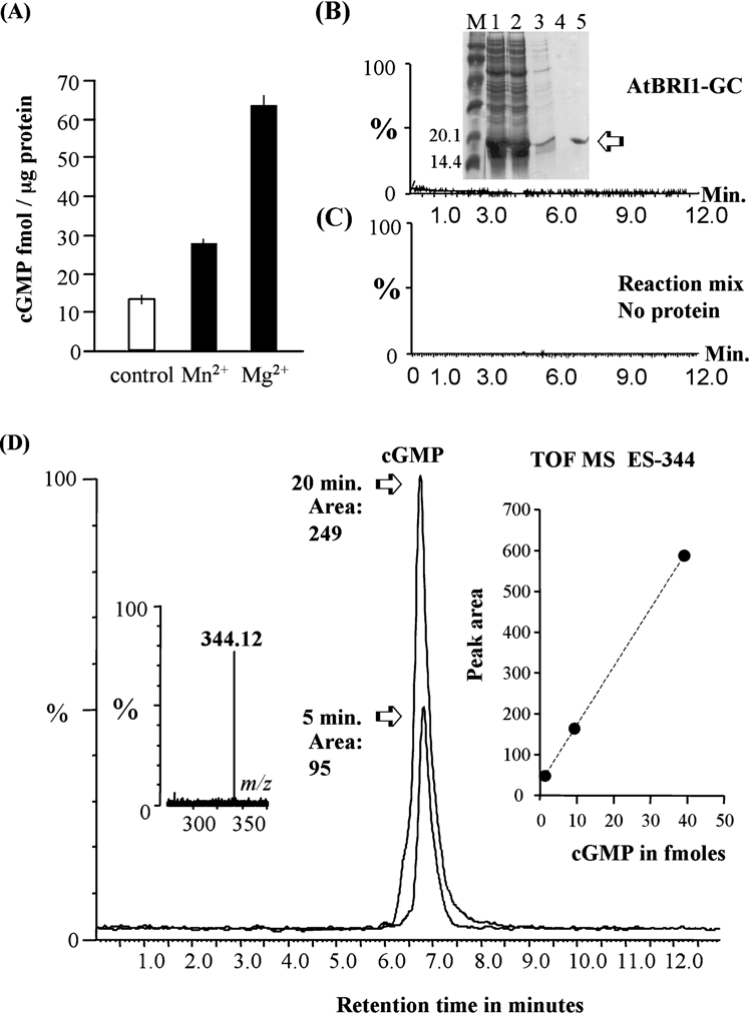
Preparation of recombinant AtBRI1-GC and functional testing *in vitro.* (A) Testing of GC activity with an enzyme immunoassay. The control contains the reaction mixture without the substrate (10 µg recombinant protein in 50 mM Tris-HCl (pH 7.5), 2 mM isobutyl methylxanthine (IBMX), 5 mM Mg^2+^ and 5 mM Mn^2+^), the other columns represent cGMP generated in the presence of 1 mM GTP and either Mn^2+^ or Mg^2+^ after 20 min. The bar values represent the mean (+/−SEM). (B) Extracted mass chromatogram of *m/z* 344 [M-1]^−1^ ion of cGMP generated by 10 µg recombinant protein. The inset shows an SDS-PAGE of AtBRI1-GC expressed in *E. coli* BL21 (pLysS) (DE3) and purified with Ni-NTA agarose under denaturing conditions. Cleared lysate (lane 1), flow through (lane 2), first wash (lane 3), second wash (lane 4) and eluted recombinant protein (lane 5). ‘M’ is the molecular weight marker. (C) Extracted mass chromatogram of *m/z* 344 [M-1]^−1^ ion of the reaction mix without AtBRI1-GC. (D) Two superimposed extracted mass chromatogram of *m/z* 344 [M-1]^−1^ ion of cGMP generated by 10 µg recombinant protein after 5 and 20 min. respectively in the presence of 5 mM Mg^2+^. (Note that the sample was diluted 200 times as compared to the experiment presented in Fig. 2A). The left inset represents the mass of the peak in the chromatogram, the right inset is the calibration curve with 1.25, 10 and 50 fmoles on the column.

The capacity of the recombinant putative GC domain of AtBRI1 (AtBRI1-GC) to generate cGMP from GTP was assessed with two independent methods. Firstly, we used an enzyme immunoassay to check if a reconstituted recombinant AtBRI1-GC could function as a GC *in vitro*. The results indicate that the recombinant protein can cyclase GTP and does so preferably in the presence of Mg^2+^ (Fig. 3A). In order to verify the result obtained with this anti-body based detection method we also used mass spectrometry. Firstly, we established that the Q-TOF mass chromatogram could detect cGMP at fmol concentrations (Fig. 3D, right inset) much like the enzyme immunoassay. We detected neither cGMP in the solution containing the recombinant protein only (Fig. 3B) nor in the reaction mix in the absence of the protein (Fig. 3C). Our sample generates cGMP in a time dependent way (Fig. 3D). We calculated that after 5 min. incubation in the presence of 1 mM GTP 100 fmoles cGMP/µg protein were generated and after 20 min. >3 pmoles cGMP/µg protein (Fig. 3D). We noted that values of the amount of cGMP generated obtained with the mass spectroscopy read higher than those obtained with the enzymatic assay and this observation has been made consistently in independent *in vitro* experiments with recombinant proteins (data not shown). In addition, it is noteworthy that plant GC activities are reportedly low and certainly not at the levels observed for some soluble animal GCs [17]. The main reason for this is that higher activities might require co-factors (e.g. Ca^2+^, chaperones or co-proteins) or post-translational modifications that are not present in the recombinant tested *in vitro*.

We also used mass spectrometry to test AtBRI1-GC for adenylyl cyclase activity in the presence of 1 mM ATP as the substrate and could not detect significant amounts of cAMP generated after 20 min. of incubation (result not shown) thus indicating that, at least *in vitro*, the recombinant protein has the predicted substrate preference for GTP.

## Discussion

Brassinosteroids (BRs) are polyhydroxylated plant steroid hormones with an essential role in co-regulating many processes including embryogenesis, cell elongation and vascular differentiation [25], [26]. Brassinosteroid Insensitive-1 (BRI1) was first identified from mutant analysis and then cloned and found to be a leucine rich repeat receptor like kinase [27] located in the plasma membrane [28], [29]. Based on the binding of the ligand BR to the leucine rich repeat extracellular domain, BRI1 has been identified as a BR receptor in Arabidopsis [29], [30] and therefore a critical signal component. BRI1 is ubiquitously expressed in Arabidopsis and potential BRI1 kinase substrates have been identified such as transthyretin-like protein which is phosphorylated *in vitro* by the kinase domain of BRI1 [31]. Several models have been developed to describe the signaling events following perception of BR by BRI1 (see [32]) involving other membrane associated proteins and activation of transcription factors. The observation that AtBRI1 does harbor a functional GC domain within the cytosolic part of the molecule might suggest that cGMP is a second messenger in some BR dependent processes. However, this hypothesis remains to be tested. Several genes that regulate physiological functions are stimulated by BR as well as being influenced by cGMP. An example for this dual dependence is plant cell elongation [26]. Microarray studies revealed that genes involved in cell wall expansion such as expansins and pectinesterases are up-regulated by both BR [32] and membrane permeable cGMP treatments [4].

Both BR and gibberellin interact to regulate plant growth. Some of these interactions are antagonistic but in other cases, BR can potentiate gibberellin activity [33]. Gibberellin, itself, stimulates increases in cGMP [7]. It is conceivable that in some instances the GC domain of BRI1 could stimulate cGMP production and so potentiate gibberellin activity. On a speculative note, there may be key molecules within specific cells that specify decreased cytoplasmic kinase activity and enhance the GC activity of the AtBRI1.

There are several recessive alleles of *AtBRI1* in the cytoplasmic kinase domain. Of these mutants, *bri-101* is the only mutant in the GC catalytic region ([E] 1078 to [L]) and it is insensitive to BR and also has reduced kinase activity when tested in a heterologous system [27], [28]. Interestingly, this mutation should not affect the GC activity as it occurs at position 8 which can be any amino acid. Three other mutants have been found in the region that we show confers GC activity *in vitro*, being: *bri-103,104* [A] 1031 to [T], *bri1-105-107* [Q] 1059 to stop (which would exclude the GC catalytic domain from the truncated protein) and *bri1-115* [G] 1048 to [N] [28]. The domain that we have identified (Fig. 2) occurs within the kinase domain [28]. We demonstrate that the isolated 114 amino acid recombinant peptide (AtBRI1-GC) has GC activity *in vitro* (Fig. 3). The relative importance of the two functions in the action of the receptor remains to be demonstrated bearing in mind that previously work has focused on the kinase domain as the GC domain had not been identified. Interestingly, a number of enzymes have recently been identified as “moonlighting” proteins with dual functions [34]; the kinase and GC activity of AtBRI1 could be yet another example.

On a more general level, the finding implies that functional GC domains may be part of a large variety of different multifunctional signaling molecules and receptors in particular. It is noteworthy that the atrial natriuretic peptide receptors NPR1 and NPR2 both signal through cGMP and have an AtBRI1-like domain organisation with an extracellular ligand-binding domain, a transmembrane domain and an intracellular kinase and GC domain [35], [36].

Finally, the fact that two recombinant proteins (AtGC1(1–100) and AtBRI1 - GC ) of less than 120 amino acids have GC activity *in vitro* begs a reexamination of the minimal catalytic requirement for GCs and may suggest that the number of different GC domains is significantly higher than currently assumed. This is in keeping with the fact that the single cellular green alga *Chlamydomonas reinhardtii* contains a surprisingly large number (>90) of annotated putative GCs [1] and with the increasing number of biological processes discovered that are modulated by the second messenger cGMP [2], [3].

## Materials and Methods

### Identification of GC catalytic domain

Annotated GCs were retrieved from NCBI and their catalytic domains [13] were used for Blast [37] queries of “The Arabidopsis Information Resource” (TAIR) database and GenBank. The catalytic domains were aligned using Clustal X [38] and the alignments at the catalytic centre of the catalytic domain was used to derive the search motifs [12]. Derived search motifs were tested for accurate and specific detection of nucleotide cyclases by querying the Protein Information Resource (www-nbrf.georgetown.edu) using the Pattern Match option on the PIR-NREF link. Search motifs were also used to query the Arabidopsis genome via the Arabidopsis server (www.arabidopsis.org) using the “Patmatch” function.

### Site-directed mutagenesis

A non-methylated double strand was synthesized using 0.5 µM Forward (5′ -ATACTGCCTATTCGATCTTCCCTTGGTGAGTGATG- 3′) and 0.5 µM Reverse (5′ – CATCACTCACCAAGGGAAGATCGAATAGGCAGTAT- 3′) primers from a clone containing the AtGC1 gene (At5g05930). The site where the mutagenesis occurred is underlined. Phusion High-Fidelity DNA Polymerase (New England Biolabs) was used in accordance with the manufactures instructions to amplify the plasmid. The original methylated template plasmid was digested using DpnI (New England Biolabs) leaving the amplified plasmid which was transformed into *E. coli* Topo 10F competent cells (Invitrogen). Single colonies were selected and the clones were analyzed by DNA sequencing.

### Synthesis of recombinant protein

Since no introns are in the putative GC domain of AtBRI1, genomic DNA from *Arabidopsis thaliana* (Col.) was used as the PCR template. PCR amplifications of *AtBRI1* GC domain (At4g39400.1: 3132–3638) (Forward primer with BamH11 site: 5′ GCTA**GGATCC**TGGAAGCTCGGGTTT 3′, reverse primer with EcoR1 site: 5′ TCCA**GAATTC**TCAAGCAACTTTTAAATGT 3′) were performed on a Mastercycler personal (Eppendorf, 22339 Hamburg Germany), in five 50 µL reaction volumes. Each reaction contained 1.5 mM MgCl_2_, 200 µM dNTPs, 0.5 µM reverse and forward primer, 7.4 ng of genomic DNA, and 2.5 units of Taq polymerase (Fermentas GmbH, St. Leon-Rot, Germany). The thermal cycling parameters were: initial denaturation at 96°C for 3 min., followed by 30 sec. at 96°C, 50°C for 45 sec. and 72°C for 1 min. for 32 cycles, followed by a final extension at 72°C for 10 min. A fragment of 340 bp was excised from the gel and purified using the GFX purification kit as per manufacturer's instruction (Amersham Biosciences, Little Chalfont, UK). The fragment was cloned into the pCR®T7 TOPO®-NT vector (Invitrogen Ltd., Paisley, UK) and used to transform *E. coli* BL21 (DE3) pLysS cells (Invitrogen Ltd., Paisley, UK); colonies were selected and inserts verified by sequencing.

### cGMP measurements

GC activity *in vitro* was assessed by measuring cGMP generated from GTP in the presence of 10 µg purified protein, 50 mM Tris-HCl (pH 7.5), 2 mM isobutyl methylxanthine (IBMX), 5 mM Mg^2+^ and/or 5 mM Mn^2+^ and 1 mM GTP [39]. Product levels were measured by cGMP enzyme immunoassay Biotrak (EIA) System (Amersham Biosciences, Little Chalfont, UK) with the acetylation protocol as described in the supplier's manual. The anti-cGMP antibody is highly specific for cGMP and has approximately 10^6^ times lower affinity for cAMP.

Mass spectroscopic determinations of cGMP were done with a Waters API Q-TOF Ultima in the W-mode. The samples were introduced with a Waters Acquity UPLC (Waters Microsep, Johannesburg, South Africa) at a flow rate of 180 µL/min. and separation was achieved by a Phenomenex Synergi (Torrance, CA) 4 µm Fusion -RP (250×2.0 mm) column. A gradient of solvent “A” (0.1% formic acid) and solvent “B” (100% acetonitrile) over 18 min was applied. During the first 7 min. the solvent composition was kept at 100% “A” followed by a linear gradient of 3 min. to 80% “B” and re-equilibration to the initial conditions. Electrospray ionisation in the negative mode was used at a cone voltage of 35 V. The running parameters were optimised for sensitivity and specificity.

## References

[1] Schaap P (2005). Guanylyl cyclases across the tree of life.. Front Biosci.

[2] Newton RP, Smith CJ (2004). Cyclic nucleotides.. Phytochem.

[3] Meier S, Gehring C (2006). Emerging roles in plant biotechnology for the second messenger cGMP - guanosine 3′,5′-cyclic monophosphate.. Afr J Biotech.

[4] Maathuis FJM (2006). cGMP modulates gene transcription and cation transport in Arabidopsis roots.. Plant J.

[5] Prado AM, Porterfield DM, Feijo JA (2004). Nitric oxide is involved in growth regulation and re-orientation of pollen tubes.. Devel.

[6] Hu X, Neill SJ, Tang Z, Cai W (2005). Nitric oxide mediates gravitropic bending in soybean roots.. Plant Physiol.

[7] Penson SP, Schuurink RC, Fath A, Gubler F, Jacobsen JV (1996). cGMP is required for gibberellic acid-induced gene expression in barley aleurone.. Plant Cell.

[8] Pharmawati M, Maryani MM, Nikolakopoulos T, Gehring CA, Irving HR (2001). Cyclic GMP modulates stomatal opening induced by natriuretic peptides and immunoreactive analogues.. Plant Physiol Biochem.

[9] Pagnussat GC, Lanteri ML, Lombardo MC, Lamattina L (2004). Nitric oxide mediates the indole acetic acid induction activation of a mitogen-activated protein kinase cascade involved in adventitious root development.. Plant Physiol.

[10] Pharmawati M, Gehring CA, Irving HR (1998). An immunoaffinity purified plant natriuretic peptide analogue modulates cGMP level in the Zea mays root stele.. Plant Sci.

[11] Donaldson L, Ludidi N, Knight MR, Gehring C, Denby K (2004). Salt and osmotic stress cause rapid increases in Arabidopsis thaliana cGMP levels.. FEBS Lett.

[12] Ludidi N, Gehring C (2003). Identification of a novel protein with guanylyl cyclase activity in Arabidopsis thaliana.. J Biol Chem.

[13] McCue L, McDonough K, Lawrence C (2000). Functional classification of cNMP-binding proteins and nucleotide cyclases with implications for novel regulatory pathways in Mycobacterium tuberculosis.. Genome Res.

[14] Liu Y, Ruoho A, Rao V, Hurley J (1997). Catalytic mechanisms of the adenyl and guanylyl cyclases: Modelling and mutational analysis.. Proc Natl Acad Sci U S A.

[15] Tucker C, Hurley J, Miller T, Hurley J (1998). Two amino acid substitutions convert a guanylyl cyclase, RetGC-1, into anadenylyl cyclase.. Proc Natl Acad Sci U S A.

[16] Tang W, Hurley J (1998). Catalytic mechanisms and regulation of mammalian adenylyl cyclases.. Mol Pharmacol.

[17] Newton R, Roef L, Witters E, Van Onckelen H (1999). Tansley Review No. 106 - Cyclic nucleotides in higher plants: the enduring paradox.. New Phytol.

[18] Kende H, Zeevaart JAD (1997). The five “classical” hormones.. Plant Cell.

[19] Chory J, Chatterjee M, Cook RK, Elich T, Fankhauser C (1996). From seed germination to flowering, light controls plant development via the pigment phytochrome.. Proc Natl Acad Sci U S A.

[20] Mussig C, Lisso J, Coll-Garcia D, Altmann T (2006). Molecular analysis of brassinosteroid action.. Plant Biol.

[21] Lindsey K, Casson S, Chilley P (2002). Peptides: new signalling molecules in plants.. Trends Plant Sci.

[22] Gehring CA, Irving HR (2003). Natriuretic peptides-a class of heterologous molecules in plants.. Int J Biochem Cell Biol.

[23] Gehring CA, Irving HR, Parish RW (1990). Effects of auxin and abscisic acid on cytosolic calcium and pH in plant cells.. Proc Natl Acad Sci U S A.

[24] Leyser O (2005). The fall and rise of apical dominance.. Curr Opin Genet Dev.

[25] Clouse SD (2002). Arabidopsis mutants reveal multiple roles for sterols in plant development.. Plant Cell.

[26] Haubrick LL, Assmann SM (2006). Brassinosteroids and plant function: some clues, more puzzles.. Plant Cell Environ.

[27] Li J, Chory J (1997). A putative leucine-rich repeat receptor kinase involved in brassinosteroid signal transduction.. Cell.

[28] Friedrichsen D, Joazeiro C, Li J, Hunter T, J C (2000). Brassinosteroid-insensitive-1 is a ubiquitously expressed leucine-rich repeat receptor serine/ threonine kinase.. Plant Physiol.

[29] Wang ZY, Seto H, Fujioka S, Yoshida S, Chory J (2001). BRI1 is a critical component of a plasma-membrane receptor for plant steroids.. Nature.

[30] Kinoshita T, Cano-Delgado A, Seto H, Hiranuma S, Fujioka S (2005). Binding of brassinosteroids to the extracellular domain of plant receptor kinase BRI1.. Nature.

[31] Nam K, Li J (2004). The Arabidopsis transethyretin-like protein is a potential substrate of BRASSINOSTEROID-INSENSITIVE 1.. Plant Cell.

[32] Goda H, Shimada Y, Asami T, Fujioka S, Yoshida S (2002). Microarray analysis of brassinosteroid-regulated genes in Arabidopsis.. Plant Physiol.

[33] Bouquin T, Meier C, Foster R, Nielsen M, Mundy J (2001). Control of specific gene expression by gibberellin and brassinosteroid.. Plant Physiol.

[34] Jeffery CJ (2003). Moonlighting proteins: old proteins learning new tricks.. Trends Genetics.

[35] Chinkers M, Garbers DL, Chang MS, Lowe DG, Chin H (1989). A membrane form of guanylate cyclase is an atrial natriuretic peptide receptor.. Nature.

[36] Garbers D, Lowe D (1994). Guanylyl cyclase receptors.. J Biol Chem.

[37] Altschul S, Madden T, Schaeffer A, Zhang J, Zhang Z (1997). Gapped BLAST and PSI-BLAST: a new generation of protein database search programs.. Nucl Acids Res.

[38] Thompson JD, Gibson TJ, Plewiak F, Jeanmougin F, Higgins DG (1997). The ClustalX windows interface: flexible strategies for multiple sequence alignment aided by quality analysis tools.. Nucl Acids Res.

[39] Thorpe S, Morkin E (1990). The carboxyl region contains the catalytic domain of the membrane form of guanylate cyclase.. J Biol Chem.

